# Avoidance of related donors in CAEBV with germline immune variants: long-termoutcome of matched unrelated donor HSCT - a case report

**DOI:** 10.1007/s00277-026-06887-4

**Published:** 2026-02-19

**Authors:** Xinran Wang, Jia Gu, Ning An, Qiuxia Yu, Yuhan Bao, Yang Gao, Jianlin Hu, Hui Luo, Chunrui Li

**Affiliations:** 1 Department of Hematology, Tongji Hospital, Tongji Medical College, Huazhong University of Science and Technology, Wuhan, 430030 Hubei China; 2 Immunotherapy Research Center for Hematologic Diseases of Hubei Province, Wuhan, 430030 Hubei China

**Keywords:** Chronic active Epstein-Barr virus infection, Germline variants, NK cell function, Hematopoietic stem cell transplantation, Matched unrelated donor

## Abstract

This study reports an adult-onset case of NK cell–predominant chronic active Epstein-Barr virus infection (CAEBV) harboring multiple heterozygous germline variants affecting antiviral immunity. Functional assessments of NK cell cytotoxicity and degranulation in the patient and her family members revealed subclinical immune defects in several relatives, leading to the exclusion of related donors. The patient ultimately underwent a fully HLA-matched unrelated donor hematopoietic stem cell transplantation (MUD-HSCT), achieving early virologic remission and complete donor chimerism. However, the post-transplant course was complicated by severe immune-related adverse events, including acute and chronic graft-versus-host disease (GVHD), thrombotic microangiopathy, viral reactivations, and secondary hemophagocytic lymphohistiocytosis, ultimately resulting in death due to severe pulmonary infection and multi-organ failure. This case underscores the critical role of immunogenetic risk stratification in guiding transplant decisions. Matched unrelated donor transplantation, supported by comprehensive functional and genetic screening, offers curative potential while avoiding the use of immunologically compromised donors. Nevertheless, long-term outcomes in CAEBV depend not only on virologic remission but also on sustained immune reconstitution. In addition, this report reviews precision transplantation strategies that integrate host genetic background, immune function, and viral dynamics, providing a roadmap for the future management of CAEBV.

## Introduction

Chronic active Epstein-Barr virus infection (CAEBV) is a rare, life-threatening lymphoproliferative disorder characterized by the clonal proliferation of EBV-infected T or natural killer (NK) cells [1]. While the pathogenesis of CAEBV remains incompletely understood, emerging evidence implicates germline mutations in genes governing immune surveillance—particularly those affecting cytotoxic lymphocyte function—as predisposing factors for disease onset and progression [2–4]. However, the clinical relevance and penetrance of many such variants remain uncertain and pose significant challenges in diagnostic and therapeutic decision-making.

Allogeneic hematopoietic stem cell transplantation (HSCT) remains the only curative therapy for CAEBV, particularly in patients with progressive disease or organ involvement [5]. Traditionally, matched related donors (MRDs) are favored for HSCT due to superior HLA compatibility and lower risk of graft-versus-host disease (GVHD) [6]. Nonetheless, emerging data suggest that MRDs may harbor undetected germline variants that compromise immune competence, potentially increasing the risk of post-transplant complications and disease recurrence [7]. In such scenarios, alternative donor strategies, including haploidentical or matched unrelated donor (MUD) transplantation, have demonstrated promising outcomes when paired with refined conditioning regimens and immune prophylaxis [8, 9].

Here, we report a clinically and genetically complex case of adult-onset NK cell–predominant CAEBV in a patient harboring multiple germline variants affecting antiviral immunity. Functional immune assessments in multiple family members revealed compromised NK cytotoxicity and degranulation, leading to the exclusion of potential related donors. The patient subsequently underwent MUD-HSCT, with initial disease control but a protracted course of immune dysregulation post-transplant. This case underscores the necessity of integrating germline genomic and functional data into donor selection algorithms and highlights the broader implications of inherited immune risk in shaping transplant outcomes.

## Materials and methods

### Ethics statement

Written informed consent was obtained from the patient and her family. The study was approved by the Institutional Ethics Review Board of Tongji Hospital, Tongji Medical College, Huazhong University of Science and Technology (Approval No. 2019S949) and was conducted in accordance with the Declaration of Helsinki.

## EBV-infected cell type identification

Peripheral blood mononuclear cells (PBMCs) were isolated using Ficoll-Paque PLUS (Cytiva, Cat# 17-1440-02) density gradient centrifugation. T cells (CD3⁺) and B cells (CD19⁺) were positively selected by magnetic-activated cell sorting (MACS) using CD3 MicroBeads and CD19 MicroBeads, respectively (Miltenyi Biotec, Cat# 130050101, 130050301). NK cells were then isolated from PBMCs by negative selection using an NK Cell Isolation Kit (Miltenyi Biotec, Cat# 130092657), according to the manufacturer’s instructions. EBV DNA levels in sorted cells, plasma and total PBMCs were quantified by real-time PCR using an EBV PCR kit (Roche, Cat# 05894943001). The predominant infected cell type was defined by a significantly elevated EBV-DNA copy number compared to unsorted PBMCs.

## Whole exome sequencing and Sanger confirmation

Genomic DNA was extracted from PBMCs and EBV-infected T/NK cells using QIAamp DNA Blood Mini Kit (Qiagen, Cat# 51106). Sequencing libraries were prepared with Illumina Nextera DNA Flex Library Prep Kit (Illumina, Cat# 20018704) and aligned to the human reference genome (hg38) using Burrows-Wheeler Aligner (BWA), and variants were called with GATK HaplotypeCaller. Variant annotation and pathogenicity prediction were conducted using CADD, REVEL, SIFT, PolyPhen-2, and MutationTaster, referencing 1000 Genomes, dbSNP, ExAC, and gnomAD databases. Sanger sequencing was performed to confirm candidate germline variants in the patient and available family members using the BigDye Terminator v3.1 Cycle Sequencing Kit (Thermo Fisher Scientific, Cat# 4337455). In addition, whole-exome sequencing (WES) of genomic DNA from whole skin tissue (without fibroblast isolation or culture) was performed as a constitutional (germline) control to distinguish germline variants from somatic variants identified in EBV-infected T/NK cells.

## NK cell cytotoxicity and degranulation assays

Freshly isolated PBMCs were cultured in RPMI-1640 (Gibco, Cat# 11875093) supplemented with 10% fetal bovine serum (Gibco, Cat# 10099141) at 2 × 10⁶ cells/mL overnight. For cytotoxicity assays, PBMCs were co-cultured with K562-EGFP cells (ATCC, Cat# CCL-243) at a 10:1 effector-to-target (E: T) ratio for 4 h. Target cell apoptosis was assessed using Annexin V-APC (BioLegend, Cat# 640920) and propidium iodide (Sigma, Cat# P4170) staining. During flow cytometric analysis, K562 target cells were identified by EGFP positivity (EGFP⁺ gate), and apoptosis/necrosis was quantified within the EGFP⁺ population. NK-cell killing activity was calculated as the percentage of Annexin V-positive and/or propidium iodide-positive EGFP⁺ target cells in co-culture (patient or control) minus the corresponding percentage in target-only wells (spontaneous/background apoptosis). The reference cutoff for normal NK-cell cytotoxicity (≥ 15.11%) was adopted from a previous study [10]. 

For CD107a degranulation, PBMCs were divided into four groups: (A) unstimulated, (B) co-cultured with K562 cells, (C) Interleukin-2 (IL-2) stimulated (100 IU/mL, PeproTech, Cat# 200-02), and (D) IL-2 stimulated + K562 co-culture. CD107a expression was measured after 4-hour incubation in the presence of monensin (Sigma, Cat# M5273) and anti-CD107a-FITC (BioLegend, Cat# 328606). Cells were stained with CD3-PerCP (BioLegend, Cat# 300328) and CD56-APC (BioLegend, Cat# 362506), and analyzed by flow cytometry. Resting and activated CD107a expression were calculated as (%CD107a_B - %CD107a_A) and (%CD107a_D - %CD107a_C), respectively.

## Results

### Clinical manifestations and diagnosis of CAEBV

A 44-year-old Han Chinese woman with no significant prior medical history presented with a two-month history of intermittent fever and persistent cough. Chest computed tomography (CT) and abdominal magnetic resonance imaging (MRI) revealed generalized lymphadenopathy. Symptomatic treatment initially resolved the fever, but relapse occurred shortly thereafter.

Quantitative polymerase chain reaction (qPCR) demonstrated elevated Epstein–Barr virus (EBV) DNA levels in plasma (7.05 × 10⁴ copies/L) and PBMCs (2.37 × 10⁶ copies/L). Cervical lymph node biopsy revealed reactive hyperplasia with EBER-positive T-cell infiltration. However, given the disease duration of less than three months and the absence of definitive histologic features of lymphoproliferation, diagnostic criteria for CAEBV were not initially met. The patient received short-term supportive management to control infection and stabilize organ function, including anti-inflammatory corticosteroids, immunomodulatory therapy, and valganciclovir as antiviral treatment. Empirical broad-spectrum antibiotics were administered for suspected bacterial infection, together with supportive measures for cardiac, hepatic, and gastric protection.

One month later, the patient was re-admitted with persistent fever. Repeat EBV testing again revealed high viral loads in plasma (1.92 × 10⁴ copies/L) and PBMCs (1.22 × 10⁶ copies/L). To determine the infected cell subset, PBMCs were sorted into T, B, and NK cell fractions using magnetic beads. Quantitative PCR analysis showed that NK cells were the predominant EBV-infected population (2.345 × 10⁷ copies/2 × 10⁵ NK cells), with comparatively lower levels in T and B cells.

At this point, the disease course had exceeded three months, accompanied by persistent fever, lymphadenopathy, elevated EBV loads in plasma and PBMCs, and EBER positivity in lymphoid tissue—fulfilling the diagnostic criteria for CAEBV with NK cell lineage predominance [6]. Extensive evaluation, including positron emission tomography (PET)-CT, bone marrow biopsy, and autoimmune and infectious panels, excluded malignancy, autoimmune disease, and other infectious etiologies. Given the clinical manifestations and laboratory findings, the patient met the hemophagocytic lymphohistiocytosis (HLH)-2004 diagnostic criteria. She fulfilled six criteria, including persistent fever; cytopenia affecting ≥ 2 lineages (hemoglobin < 90 g/L and platelet count < 100 × 10⁹/L); hyperferritinemia (ferritin ≥ 500 µg/L); hypofibrinogenemia (fibrinogen < 1.5 g/L); and hemophagocytosis in the bone marrow. In addition, NK-cell cytotoxic activity was markedly reduced. Accordingly, HLH was diagnosed **s**econdary to CAEBV.

## Genetic and immunologic evaluation

Whole exome sequencing (WES) for skin tissue and NK cells was performed to screen germline and somatic mutations of this patient. We identified 7 heterozygous germline variants in 7 genes (*ZAP70 p.Arg104Gln*,* CD247 p.Gly109Cys*,* TYK2 p.Arg221Trp*,* TMEM173 p.Arg220His*,* MEEV p.Ser749Tyr*,* TCF3 p.Lys522Gln*,* IL12RB1 p.Arg156His*), which belonged to the set of the 372 anti-viral immunity-associated genes conducted in our previous study [11]. Three germline variants were identified in genes previously reported to be associated with EBV infection, including two EBV-related genes (*ZAP70* and *TYK2*) [12, 13] and one gene involved in T/NK-cell function (*CD247*) [14]. All variants were further confirmed in the patient’s family by Sanger sequencing (Table 1). On the other hand, we found somatic variants in *DDX3X*, *KMT2D*, and *ARID1A* in EBV-infected NK cells, which have been recurrently reported in EBV-associated T/NK lymphoproliferative diseases with poor prognosis [11, 15].

**Table 1:** Mutations and NK-cell cytotoxic function of patient’s family members

Family members	Germline variants			Somatic mutation in NK cells	EBV infection	NK-cell killing activity	Resting CD107a level	Activated CD107a level
	*ZAP70* *p*.Arg104Gln	*CD247 p*.Gly109Cys	*TYK2 p*.Arg221Trp					
Patient	+	+	+	DDX3X: p.T498A; KMT2D: p.2031_2037del, p.Arg3707Ter; ARID1A: p.Gln269Ter	+ (NK)	3.76%	5.60%	22.90%
Father	-	-	+	/	-	8.65%	39.42%	84.56%
Mother	+	+	-	/	-	17.09%	35.24%	78.20%
Brother 1	-	-	-	/	+ (B)	7.33%	36.03%	71.99%
Brother 2	-	-	+	/	-	15.33%	8.54%	33.49%
Sister	-	-	+	/	-	23.08%	5.29%	17.20%
Daughter	-	+	-	/	-	4.80%	21.77%	42.82%
Son	+	+	+	/	-	5.92%	11.12%	17.72%
Cousin	/	/	/	/	-	17.37%	19.29%	57.29%

All germline variants detected in the individuals were heterozygous

**Fig. 1:**
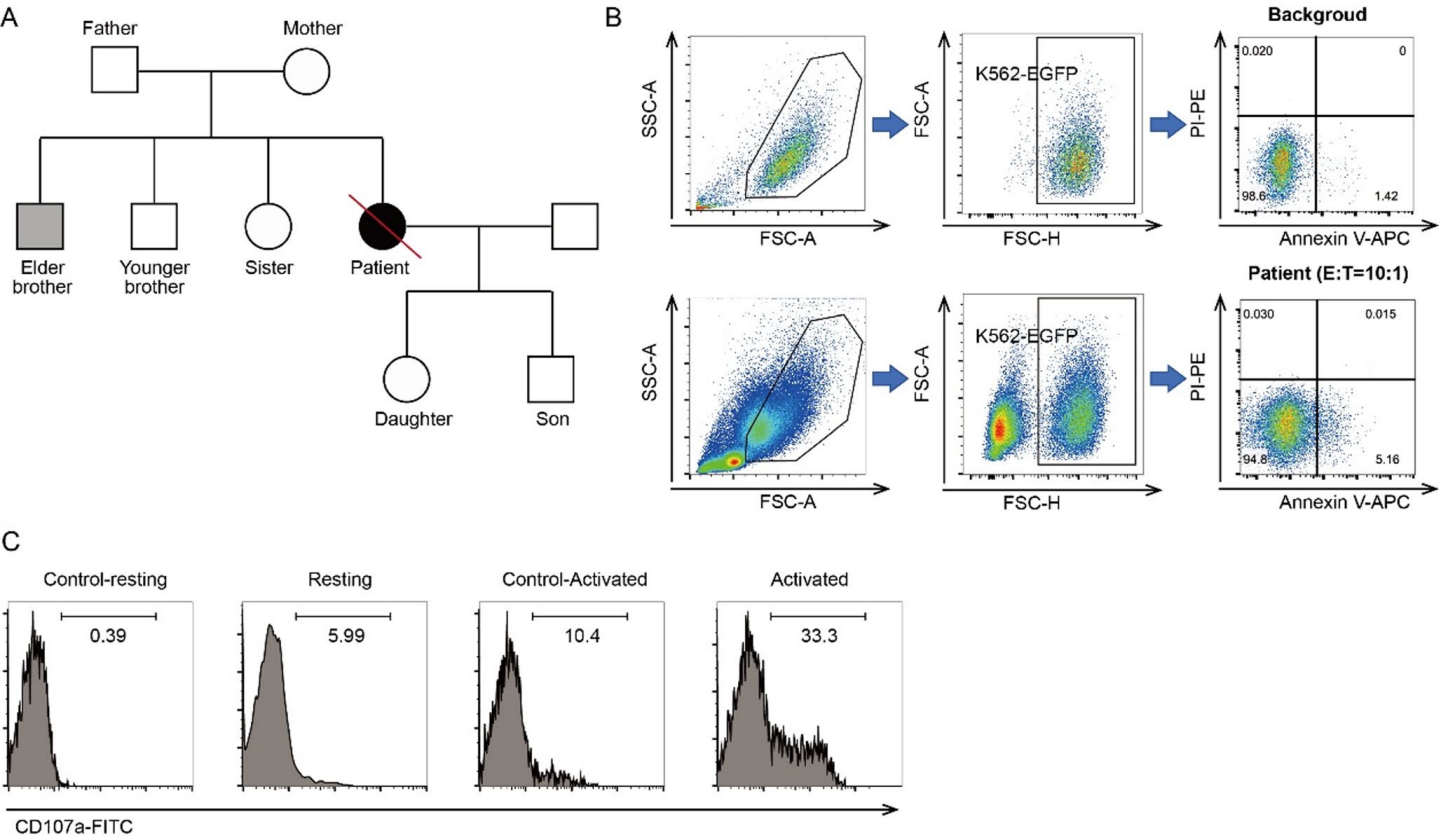
Functional Impairment of NK Cells in the Patient with CAEBV. (**A**) Pedigree chart illustrating the proband (solid black symbol) and family members evaluated for EBV infection and immune function. The elder brother (grey symbol) exhibited chronic EBV infection in B cells accompanied by reduced NK cell cytotoxicity. (**B**) NK cell cytotoxicity was assessed by flow cytometry at an effector-to-target (E:T) ratio of 10:1. Cells were gated by FSC-A vs SSC-A, and then K562-EGFP target cells, followed by Annexin V/PI analysis. Annexin V/PI staining shows the percentage of early apoptotic and necrotic target cells in the patient (coculture of PBMCs and K562-EGFP) compared with the background control (untreated K562-EGFP cultured alone) (**C**) CD107a degranulation assay indicates markedly diminished NK cell activity in the patient. Resting and activated CD107a expression were calculated as (%CD107a_Resting - %CD107a_Control-resting) and (%CD107a_Activated - %CD107a_Control-Activated), respectively. Data represent the percentage of CD107a⁺ cells within the NK cell population

NK cell cytotoxicity assays were performed to assess the functional consequences of the identified mutations. The patient exhibited markedly reduced NK cell killing activity (3.76%, normal ≥ 15.11%), as well as reduced expression of CD107a in NK cells following IL-2 stimulation (22.90%, normal ≥ 40%) (Fig. 1B-C). NK functional assays in her family members were also conducted. The data showed that her father, elder brother and her two children had obvious reduction of NK cell killing activity, and younger brother, sister and son had reduced expression of activated CD107a in NK cells. Her non-direct relative, cousin, had completely normal NK cell killing activity and CD107a expression in NK cells. In addition, her elder brother had a chronic EBV infection involving B cells (Fig. 1A).

## Pre-transplant disease course and rationale for choosing MUD-HSCT

Initial management with low-dose dexamethasone for over one month yielded suboptimal response, with EBV DNA levels in PBMCs increasing to 6.84 × 10⁶ copies/L. The patient subsequently received six cycles of etoposide (100 mg per cycle) over a four-month period, which failed to achieve virologic or clinical remission. PBMC EBV DNA decreased only marginally to 2.51 × 10⁶ copies/L, and plasma EBV DNA to 1.76 × 10³ copies/L.

Given persistent active disease and progression, HSCT was pursued. Functional NK cell profiling and germline genetic screening revealed multiple family members carried variants associated with impaired cytotoxicity or degranulation (Table 1), raising concerns about using related donors. Consequently, a 10/10 HLA-matched unrelated donor was selected from the national bone marrow registry to avoid the risk of transferring subclinical immunodeficiency or latent EBV susceptibility.

### Transplantation procedure and post-transplant course

Seven months after initial diagnosis, the patient underwent myeloablative conditioning using etoposide, busulfan, cyclophosphamide, and anti-thymocyte globulin/anti-lymphocyte globulin (ATG/ALG), followed by infusion of 3.38 × 10⁶/kg CD34⁺ cells. GVHD prophylaxis consisted of cyclosporine A. Neutrophil engraftment was achieved by day 12, and complete donor chimerism was confirmed (CD3⁺: 97%, CD3⁻: 100%).

**Fig. 2:**
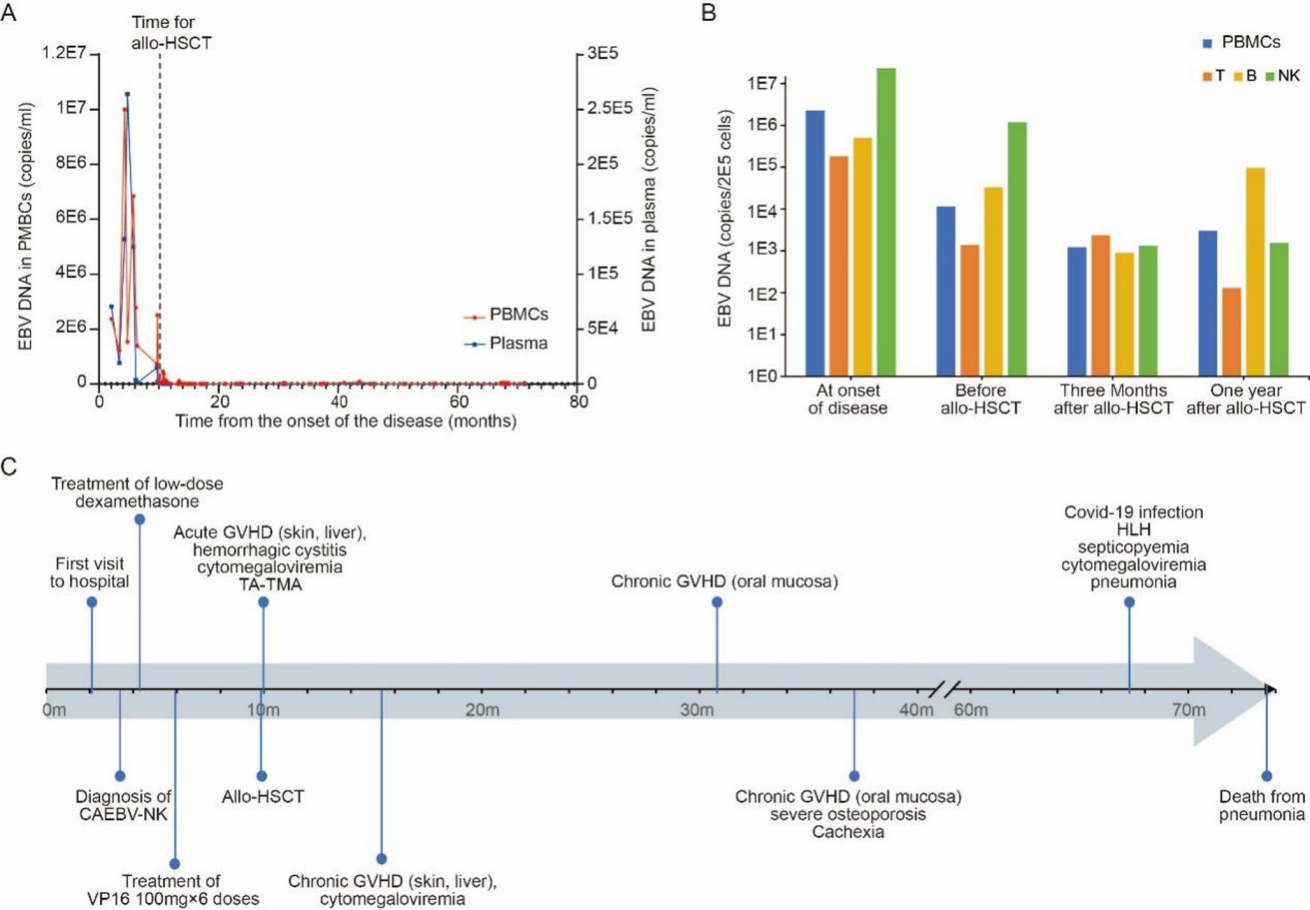
Longitudinal EBV Kinetics and Clinical Course in a Patient with CAEBV. (**A**) Serial quantification of EBV DNA in PBMCs (red) and plasma (blue) over the disease course. EBV load peaked during the early phase of the disease and declined dramatically following allo-HSCT (vertical dashed line), with persistently low-level viremia in PBMCs and undetectable plasma EBV DNA thereafter. (**B**) EBV-infected cell subsets at four critical timepoints, assessed by cell-sorting PCR, revealed a shift from NK-cell predominance before transplantation to B-cell predominance after HSCT, indicating dynamic changes in viral reservoirs. (**C**) Timeline of clinical events from initial diagnosis to post-transplant complications and outcome. Key phases included corticosteroid and VP-16 (etoposide) therapy, allo-HSCT at month 7, acute and chronic GVHD, infectious complications, and eventual death from pneumonia associated with coronavirus disease 2019 (COVID-19) and cytomegalovirus (CMV) reactivation at 63.7 months post-HSCT

Following transplantation, EBV DNA levels in PBMCs dropped rapidly and remained low. Plasma EBV DNA became undetectable (Fig. 2A). EBV-targeted cell sorting PCR at 3 and 12 months revealed a shift in EBV tropism from NK cell to B cell predominance (Fig. 2B), consistent with viral clearance in the primary lineage. The patient was thus considered to have achieved complete remission.

Post-transplant donor chimerism was reassessed at additional time points beyond d + 12 to monitor engraftment. At d + 30, d + 60, and d + 90, donor chimerism remained stable in both fractions (CD3⁺: 95–98%; CD3⁻: 100%), supporting sustained donor engraftment and donor hematopoiesis. These findings were consistent with the engraftment documented at d + 12 and indicated ongoing stable donor-derived hematopoiesis.

Despite virologic remission, the post-transplant course was complicated. Acute skin and liver GVHD developed early post-HSCT and required stepwise immunosuppressive adjustment from methylprednisolone and cyclosporine to tacrolimus, sirolimus, and ruxolitinib due to steroid-induced vertebral compression fractures. The patient also experienced grade 3 mucositis, CMV reactivation, and hemorrhagic cystitis.

At 1.7 months post-HSCT, she was diagnosed with transplant-associated thrombotic microangiopathy (TA-TMA), presenting with thrombocytopenia, jaundice, and 10% schistocytosis. Discontinuation of tacrolimus and initiation of plasma exchange and ruxolitinib achieved clinical stabilization.

Twenty-one months post-HSCT, the patient developed extensive oral chronic GVHD, which persisted for over six months and led to severe dysphagia and cachexia. Long-term tacrolimus and corticosteroids were required. At 57.4 months, she was admitted with COVID-19 pneumonia complicated by CMV reactivation and secondaryHLH, from which she gradually recovered. However, at 63.7 months post-transplant, she succumbed to severe pulmonary infection and multi-organ failure (Fig. 2C).

## Discussion

CAEBV is a rare, life-threatening lymphoproliferative disorder driven by the persistent clonal expansion of EBV-infected T or NK cells. Although allogeneic hematopoietic stem cell transplantation (allo-HSCT) remains the only curative treatment, CAEBV is biologically heterogeneous, involving underlying germline susceptibility, immune dysfunction, and dynamic virus–host interactions. This case of adult-onset NK cell–predominant CAEBV with multiple heterozygous germline variants in *ZAP70*, *CD247*, and *TYK2* exemplifies the need for precision-guided approaches across diagnosis, donor selection, transplantation, and post-HSCT management.

### Germline and somatic immune profiling in CAEBV

Our patient harbored germline variants in *ZAP70* (p.Arg104Gln), CD247 (p.Gly109Cys), and *TYK2* (p.Arg221Trp), all key regulators of antiviral immunity. *ZAP70* is essential for TCR signaling and T/NK cell development; hypomorphic variants impair cytotoxic function and have been associated with EBV-driven immunodeficiency [16]. *CD247* encodes the CD3ζ chain and mediates signal transduction in both TCR and NK receptor complexes; the p.G109C variant may disrupt structural integrity and impair NK degranulation [17]. TYK2 is a Janus kinase involved in type I interferon signaling, and the p.R221W variant likely compromises antiviral cytokine responses [18]. The presence of these multiple heterozygous germline variants suggests a multiple-hit immune susceptibility background, predisposing to severe CAEBV and potentially influencing immune recovery after transplantation.

In experimental systems, selected* ZAP70* mutants with impaired kinase activity can suppress TCR signaling despite the presence of a wild-type allele, which has been interpreted as dominant-negative interference in specific settings [19]. Similar inhibitory effects on downstream signaling have also been reported for kinase-deficient TYK2 constructs in defined experimental contexts [20]. These observations provide biological plausibility for allele-interfering mechanisms. However, they are model-dependent and do not establish a dominant-negative mechanism for the naturally occurring heterozygous variants identified in our patient. In our case, no functional validation was performed to test this hypothesis, and the clinical relevance therefore remains uncertain.

In addition to germline variants, somatic mutations in *DDX3X*, *KMT2D*, and *ARID1A* were identified in EBV-infected NK cells. These mutations are recurrently reported in EBV-associated T/NK-cell lymphoproliferative disorders and are implicated in epigenetic dysregulation and resistance to immune surveillance. Together, these germline and somatic alterations reveal a complex immune landscape that influences disease course and transplant outcomes.

Regarding the two somatic *KMT2D* variants (p.2031_2037del and p.Arg3707Ter), phasing could not be resolved using the available short-read sequencing data. Therefore, it remains uncertain whether these variants are in trans (compound heterozygous) or in cis, and whether they occur within the same clone. The variant allele frequencies (VAFs) differed (0.078 vs. 0.131), which is compatible with the possibility of distinct subclones. Further resolution would require additional approaches such as long-read sequencing or single-cell analyses, which were not performed.

### Donor selection in genetically predisposed families

While HLA-matched related donors are traditionally preferred for allo-HSCT, this assumption warrants caution in CAEBV, particularly in familial or adult-onset cases. In our case, functional assays revealed impaired NK cell cytotoxicity and/or reduced CD107a degranulation in some clinically healthy first-degree relatives, suggesting subclinical immune variation within the family. Using such donors risks transmitting defective immune function and may increase post-transplant complications. A 10/10 MUD was thus selected from the national registry, which enabled early EBV control and successful engraftment without the confounding effects of inherited immune defects. These findings support emerging evidence that MUD-HSCT, when combined with immunogenetic profiling, can yield survival outcomes comparable to MRD-HSCT in CAEBV.

### Immune reconstitution challenges and post-transplant vulnerabilities in CAEBV

Despite achieving full donor chimerism and early EBV clearance, the patient experienced multiple severe complications, including acute and chronic GVHD, thrombotic microangiopathy (TMA), viral reactivations, and secondary HLH. This disconnects between virologic remission and immune dysfunction underscores the limitations of traditional surrogate endpoints such as EBV load and chimerism [21–23].

Notably, our patient exhibited poor prognostic features described in CAEBV cohorts, namely age ≥ 15 years at diagnosis and elevated soluble IL-2 receptor (sIL-2R) levels [24–26], which correlate with worse outcomes after HSCT. These likely reflect persistent immune dysregulation not fully corrected by transplantation. These likely reflect persistent immune dysregulation not fully corrected by transplantation.

Moreover, EBV-infected cells shifted from NK to B cell predominance after transplant, suggesting the potential for viral persistence in alternative reservoirs. This phenotypic shift raises concern for post-HSCT relapse or latent reactivation and highlights the need for continued immune surveillance beyond standard viral PCR.

### Genetic and environmental factors in immune dysregulation of CAEBV

The family observations in this case do not support a simple causal relationship between the identified heterozygous germline variants and NK-cell dysfunction. Instead, these variants are best considered potential susceptibility factors with incomplete penetrance and variable expressivity. The elder brother, despite not carrying the reported variants, showed reduced NK cytotoxicity in the setting of B-cell–predominant chronic EBV infection, suggesting additional contributors beyond the variants identified here. These may include genetic factors not captured by our approach (e.g., variants outside coding regions or in other loci), as well as non-genetic influences [27, 28]. Conversely, the mother’s normal NK-cell killing and degranulation despite carrying two variants is compatible with compensatory immune mechanisms and phenotypic variability among individuals with similar genetic backgrounds [29–31]. Taken together, these findings highlight the multifactorial nature of immune dysregulation in CAEBV and the limitations of inferring causality from observations within a single family.

### Toward risk-adapted transplant strategies

Management of CAEBV must move toward biologically defined risk stratification. Patients with early diagnosis, younger age, and no immune gene mutations may benefit from reduced-intensity conditioning (RIC) and hematopoietic stem cell transplantation from either matched related donors or matched unrelated donors. In contrast, high-risk patients characterized by germline mutations, impaired NK or T cell function, or systemic inflammation may require myeloablative conditioning (MAC), intensified GVHD prophylaxis, and pre-transplant immunomodulatory bridging therapy such as ruxolitinib or L-DEP to reduce disease burden and cytokine-mediated toxicity [32, 33]. Furthermore, the emergence of B cell EBV reservoirs post-HSCT supports the integration of adoptive immunotherapies such as EBV-specific T cells for long-term viral control and relapse prevention [34, 35].

### Precision transplantation in CAEBV: future directions

This case underscores several critical gaps and future directions in CAEBV management. First, germline and somatic immune profiling—including functional validation of high-risk variants—should become routine in pre-transplant evaluation. Second, donor selection should extend beyond HLA typing to include assessment of NK and T cell cytotoxicity. Third, immune recovery post-HSCT must be longitudinally tracked using functional and molecular biomarkers (e.g., cytokine panels, EBV reservoir mapping) to anticipate and prevent complications like GVHD, HLH, and infections. Lastly, novel personalized immunotherapeutics-such as checkpoint inhibitors, IL-1/IL-6 antagonists, and virus-specific T cells-should be considered in patients with residual immune dysregulation or mixed-lineage viral persistence.

## Conclusion

This case of NK cell–predominant CAEBV with multiple heterozygous germline immune variants illustrates the critical role of immunogenetic risk stratification in guiding transplant decisions. Matched unrelated transplantation, supported by functional and genetic screening, can provide curative potential while avoiding immunologically compromised donors. However, long-term outcomes in CAEBV depend not only on virologic remission but also on sustained immune reconstitution. Integrating host genetics, immune function, and viral dynamics defines the path forward for precision transplantation in CAEBV.

## Data Availability

The datasets used and analyzed during the current study are available from the corresponding author upon reasonable request.
